# Recent Advances in Molecularly Imprinted Polymers and Their Disease-Related Applications

**DOI:** 10.3390/polym15214199

**Published:** 2023-10-24

**Authors:** Celia Cabaleiro-Lago, Sylwia Hasterok, Anette Gjörloff Wingren, Helena Tassidis

**Affiliations:** 1 Department of Bioanalysis, Faculty of Natural Sciences, Kristianstad University, 291 39 Kristianstad, Sweden; celia.cabaleiro_lago@hkr.se (C.C.-L.); helena.tassidis@hkr.se (H.T.); 2 Department of Biomedical Sciences, Faculty of Health and Society, Malmö University, 205 06 Malmö, Sweden; sylwia.hasterok@mau.se; 3 Biofilms-Research Center for Biointerfaces, Malmö University, 205 06 Malmö, Sweden

**Keywords:** biomarker, diagnostics, disease, medical applications, molecularly imprinted polymers, sensor

## Abstract

Molecularly imprinted polymers (MIPs) and the imprinting technique provide polymeric material with recognition elements similar to natural antibodies. The template of choice (i.e., the antigen) can be almost any type of smaller or larger molecule, protein, or even tissue. There are various formats of MIPs developed for different medical purposes, such as targeting, imaging, assay diagnostics, and biomarker detection. Biologically applied MIPs are widely used and currently developed for medical applications, and targeting the antigen with MIPs can also help in personalized medicine. The synthetic recognition sites of the MIPs can be tailor-made to function as analytics, diagnostics, and drug delivery systems. This review will cover the promising clinical applications of different MIP systems recently developed for disease diagnosis and treatment.

## 1. Introduction

The field of medicine and biotechnology is in constant need of novel applications and the development of immunoassays, biosensors, imaging techniques, and other methods that can provide detailed information about targeting for disease diagnosis and treatment.

Molecularly imprinted polymers (MIPs) are “antibody mimics” made from the co-polymerization of functional and cross-linking monomers around the “antigen” of interest or a derivative thereof, called the “template” [1]. The design of MIPs and their use as artificial recognition elements have successfully targeted a variety of molecules by mimicking recognition events similar to those in biological recognition processes [2]. The target molecule, the template, can be a protein, peptide, lipid, amino acid, virus, cell, nucleic acid, or even a more complex glycan structure [3]. The molecular imprinting technique was first reported in 1972 by Wulff and Sarhan [4] and has since been adopted by researchers. Facilitated by the hybridization of material from the two research fields of science and biology, recent research in the field has developed and utilized various polymerization synthesis methods tailored to the biological application of MIPs [5]. MIPs have numerous biological applications, such as immunoassays, including the enzyme-linked immunosorbent assay (ELISA) and immune-affinity separation; optical and electrochemical sensors; imaging; and drug delivery. MIPs have been comprehensively reviewed in research, including aspects such as the different synthetic approaches; the wide range of monomers, cross-linkers, and initiators; and the type of interaction between the template and polymer. Since the chemical development and analyses of the synthesized MIPs to a large extent already have been reviewed, the novelty of this review is the medical focus on the most common biomarkers for certain diseases and the potential usage of MIPs in biosensors and drug delivery.

**Figure 1:**
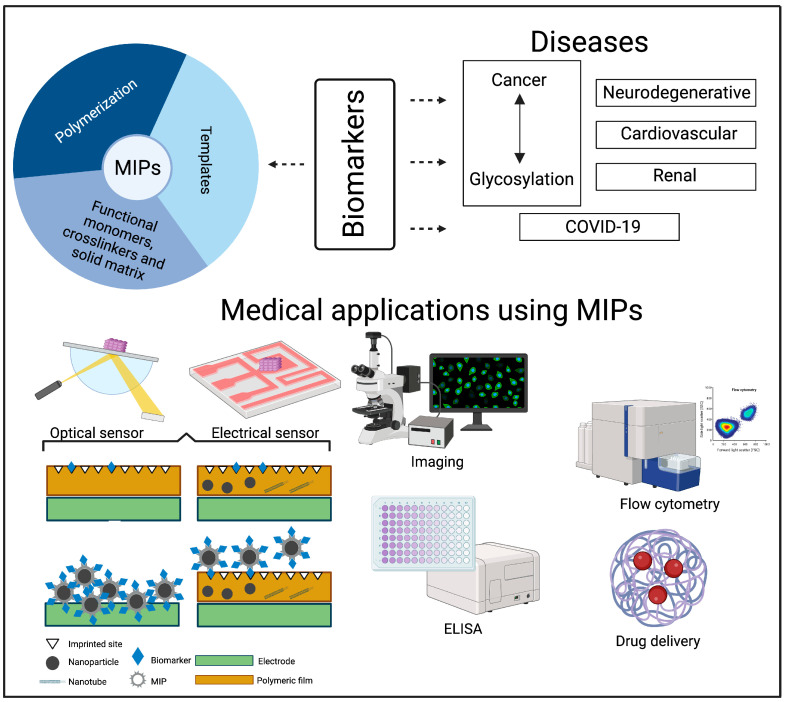
The MIP imprinting technique includes several components and steps that should be carefully selected for medical applications. The biomarkers representing the template of interest depend on the disease, as well as on the chosen application. The MIP systems developed and described in this review include the diagnosis or treatment of the following diseases: cancer (particularly focusing on glycosylation, drug delivery, and biosensors), neurodegenerative disorders, cardiovascular diseases, renal diseases, and COVID-19. The most common applications of MIP systems within the medical domain are optical and electrical sensors, imaging, flow cytometry, and ELISA. In the case of sensors, four different formats for MIP sensors are presented: polymeric film on electrode, polymeric film with nanomaterials on electrode, MIP deposited on electrode, and polymeric film with nanomaterials on electrode combined with MIPs.

The aim of this review is to discuss the latest advancements in MIP systems and their practical use in detecting a wide range of diseases (Figure 1). We begin with an overview of the fundamental principles behind the MIP imprinting technique. Subsequently, we delve into the diverse applications of MIP systems within the medical domain, with a focus on targeting and biomarker detection. Finally, we engage in an extensive discussion concerning the integration of MIP systems for the diagnosis of various diseases, including cancer (particularly focusing on glycosylation, drug delivery, and biosensors), neurodegenerative disorders, cardiovascular diseases, COVID-19, and renal diseases (Figure 1). An important field within medicine is the use of MIPs for the detection of pharmaceuticals (e.g., therapeutic drug monitoring), but this will not be covered in this paper since comprehensive information can be found elsewhere [6,7,8].

## 2. MIPs and Imprinting Techniques

The rationale behind MIPs is straightforward: the MIP synthesis process relies on functional monomer(s) that form stable interactions with a template molecule [5]. The stability and specificity of the template–monomer complex is crucial for the recognition of the imprinted material. A cross-linker that creates a 3-D polymeric structure around the template–monomer complex is added together with a solvent or porogen. Afterwards, polymerization is initiated by diverse mechanisms. The final step is the removal of the template, which results in a material with imprinted cavities specific to the template. Molecular imprinting can be divided into covalent and non-covalent imprinting based on the type of interaction between the template and the functional monomers. Non-covalent imprinting is commonly used for most MIP applications due to its greater flexibility regarding the types of monomers and templates [9].

Different polymerization approaches are currently used to prepare MIPs. One of the first and most utilized approaches is bulk polymerization, which results in a monolithic material that needs to be ground for further use. This approach produces irregular particles with low or variable accessibility to the binding sites. The monolithic approach in situ is successfully used to prepare chromatographic materials directly on a chromatographic column [10]. Great effort has been directed towards preparing MIPs with a controllable size. This has led to the development of other polymerization approaches, such as suspension, emulsion, or Pickering emulsion [11], whereas precipitation [12] is among the less common approaches, together with multi-swelling and sol–gel polymerization [13]. Solid-phase synthesis is another method that provides even-sized imprinted nanoparticles with good accessibility and affinity to the sites and easy preparation and scalability [14,15]. These approaches have been combined with different types of polymerization reactions, such as free radical polymerization and controlled radical polymerization [16]. Another way to create imprinted materials is surface imprinting by grafting surfaces or porous particles. This approach provides easier access to the binding sites and is usually preferred for templates of great size, such as macromolecules and proteins [17]. Electropolymerization is a special type of polymerization in which a thin imprinted film is polymerized directly on a conductive electrode surface [18].

Classical methods for MIP synthesis deal with procedures that minimize the contact with water between the monomers, initiator, and template, which often results in non-water-soluble MIP particles. This can be advantageous in applications such as chromatography and solid-phase extraction, but applications of MIPs as drug delivery systems require a water-compatible MIP [19]. Hydrophilic templates present a challenge regarding the type of porogen. Mainstream MIP synthesis uses aprotic organic solvents that maximize the interactions between the functional polymer and the template, allowing higher affinity between the MIP and the template. However, hydrophilic templates are poorly soluble in these solvents. Moreover, although polar templates can successfully be imprinted in polar MIPs, the performance of the MIPs can still be poor when used in aqueous media. An alternative approach to this challenge is to use, for example, hydrophilic polymers (which implies fewer available functional monomers) or the post-modification of apolar MIPs [16].

Imprinting high-molecular-weight compounds, such as bio-macromolecules (e.g., proteins), carries additional challenges for the preparation of MIPs. These challenges include the complexity of the structure and conformation; the size and the flexibility of the target molecule, which may lead to low affinities and heterogeneous binding sites; the poor diffusion of the target through the MIP; and even the poor removal of the template after imprinting [15,20]. In most cases, the MIPs used for these purposes must be prepared, i.e., synthesized and kept stable in aqueous solutions. Hence, this limits the method of preparation and the types of monomers and cross-linkers. A way to overcome this problem is to perform MIP synthesis in organic solvents and use specific epitopes of the target instead of whole proteins or macromolecules, which may be affected by the apolar solvent [15,21]. Solid-phase polymerization is a promising technique that can yield single-protein-imprinted monodisperse MIPs and is widely used for the imprinting of proteins [15]. In this approach, the target molecules are covalently attached to support beads. The polymerization is then started in order to create monodisperse MIP particles around each immobilized target.

MIPs designed for imaging have a new challenge: they have to emit a detectable signal—for example, a fluorescence signal. Different approaches have been used here, including polymerizable fluorophores, fluorophore-doped MIPs, surface-imprinted particles (quantum dots or carbon dots) with inherent luminescence signals [22], and Raman active nanoparticles [23].

## 3. MIP Technology and Medical Applications

MIPs have long been described as a promising alternative to antibodies for biochemical and biomedical applications. The great specificity and sensitivity of antibodies against an antigen can be achieved with MIP technology. Moreover, MIPs surpass antibodies regarding chemical and physical stability, allowing long-term storage and reusability at a lower cost. MIPs can be designed to bind to a diverse range of molecules, including those that natural antibodies do not recognize—for example, polysaccharides [1] or phospholipids [24]. On the other hand, developing MIPs for a specific target requires a tedious optimization process where no general protocol for synthesis is available, and the repeatability between different MIP batches may be low. Other common drawbacks of MIPs are the heterogeneity of the imprinted cavities and template bleeding [1,23].

Extraction and separation from complex matrices with MIPs have been a central research topic since the technique was developed. Successful materials have been developed for sample preparation prior to analytical measurements. This is, in fact, one of the few commercial applications of MIPs. MIPs can capture specific molecules in complex matrices, such as environmental molecules and bio-fluids. MIPs have been explored as systems for the extraction of diverse biomarkers, from small metabolic molecules to larger protein biomarkers. A biomarker shows a specific physical trait or a measurable biologically produced change in the body that is linked to a disease or a health condition. Some biomarkers have a low concentration in the matrix, and purification and pre-concentration aided by MIPs can lead to more sensitive analytical detection [25]. Indeed, MIPs could help to solve the need for fast, cost-effective, and sensitive analytical methods.

MIP-based sensors are currently used as a cost-effective approach to design synthetic recognition sites for various substances, from environmental pollutants to pharmaceuticals. Optical sensors measure the change in the optical characteristics of the transducer surface when the target and recognition site forms a complex, whereas electrochemical sensors serve as smart devices for electrochemical output [26,27]. Comprehensive reviews on sensor construction and performance can be found elsewhere [28,29,30]. The ease of adapting MIPs in sensors has increased the practical applications in many fields. Recent developments in biotechnology can provide sensors that are more effective, highly selective, and sensitive. Other advantages are long-term stability and reusability, low costs, and ease of preparation [31]. Research indicates that the large-scale production of MIPs is cheaper than the preparation of antibodies [14,23]. Therefore, MIP-based sensors have wide prospects for the detection of biomolecules in medical diagnostics, as well as for the determination of pharmaceuticals such as antibiotics [32,33]. Nonetheless, challenges have to be overcome before the commercial use of MIP-based sensors. Fouling problems are common, and template stripping after use is not trivial. Moreover, there is broad variation in materials and procedures but no standard protocol for preparation, which can lead to problems in reproducibility [34].

Drug delivery systems (DDS) comprehend the process of the body to achieve a desired therapeutic effect. The goal of drug delivery is to administer therapeutic substances in a controlled manner to a specific site and achieve the maximum therapeutic benefit while minimizing side effects and toxicity. MIPs can exhibit high selectivity, and the recognition sites have exactly the same characteristic chemical properties as the template molecule, making them a good candidate for effective drug delivery [35]. When a drug is loaded into the MIP, it binds specifically to the imprints, resulting in controlled drug release over time. The drug can be released by changing the pH, the temperature, or the ionic strength of the environment. One challenge with MIPs is that they often require a high degree of cross-linking to maintain their conformation when the template molecule is absent, but they allow the easy binding and release of the target molecule. The nature of the solvent is also important to consider, and it becomes highly relevant when conducting in vivo studies. Firstly, minimizing toxicity based on the solvent ideally requires the synthesis to be performed in aqueous media. Secondly, MIPs as DDS should function in aqueous media; therefore, synthesis in water or other protic and polar solvents is preferred [36]. Other challenges in using MIPs as DDS are biocompatibility and biodegradability. MIPs used for drug delivery usually show biocompatibility when biocompatible polymers are used, but long-term toxicology studies have not been performed [37]. Furthermore, protein corona formation in biological fluids should be considered. As with nanoparticles [38], the protein corona is a key factor for the distribution, function, and clearance of MIP DDS [39].

## 4. MIPs and Disease

MIP applications in biomedicine span diverse diseases. One of the most common fields of research for MIPs is cancer, but applications can also be found in relation to cardiovascular and neurodegenerative diseases. MIP applications in other disease diagnoses are discussed briefly in the following section with a focus on biosensing.

### Cancer

The diagnosis and disease progression of cancer are dependent on the development of new and efficient sensors or assays to identify biomarkers in clinical tumor samples. Molecular imprinting technology possesses high specificity and selectivity in chemical recognition, comparable to antibodies. MIPs intrinsically have low costs, high stability, and versatility.

(a) MIPs targeting glycosylation in cancer

Polysaccharides, or glycans, are carbohydrate-based polymers linked to almost all proteins or lipids [40]. Monosaccharides are the basic units of glycans. Targeting glycans and glycosaminoglycans with antibodies or lectins is challenging, due to the lack of specificity and stability. Instead, MIPs have been used as an alternative [3,41,42,43,44,45,46,47,48,49]. Glycosylation has an important role in cancer biology and disease progression [50], which makes the monosaccharide sialic acid (SA) a versatile biomarker for many cancer types. Several research groups have explored and reviewed glycosylation [1,3,51], specifically SA-imprinted MIPs, in cancer-associated applications for diagnostics. One interesting approach reviewed by Ali et al. [51] is the development of nano-MIPs against the immune checkpoint inhibitor ligand programmed death-ligand 1 (PD-L1). Inhibiting the signaling pathways between this receptor–ligand pair involving a reactivate T-cell response has proven to be an effective cancer therapy technique.

**Figure 2:**
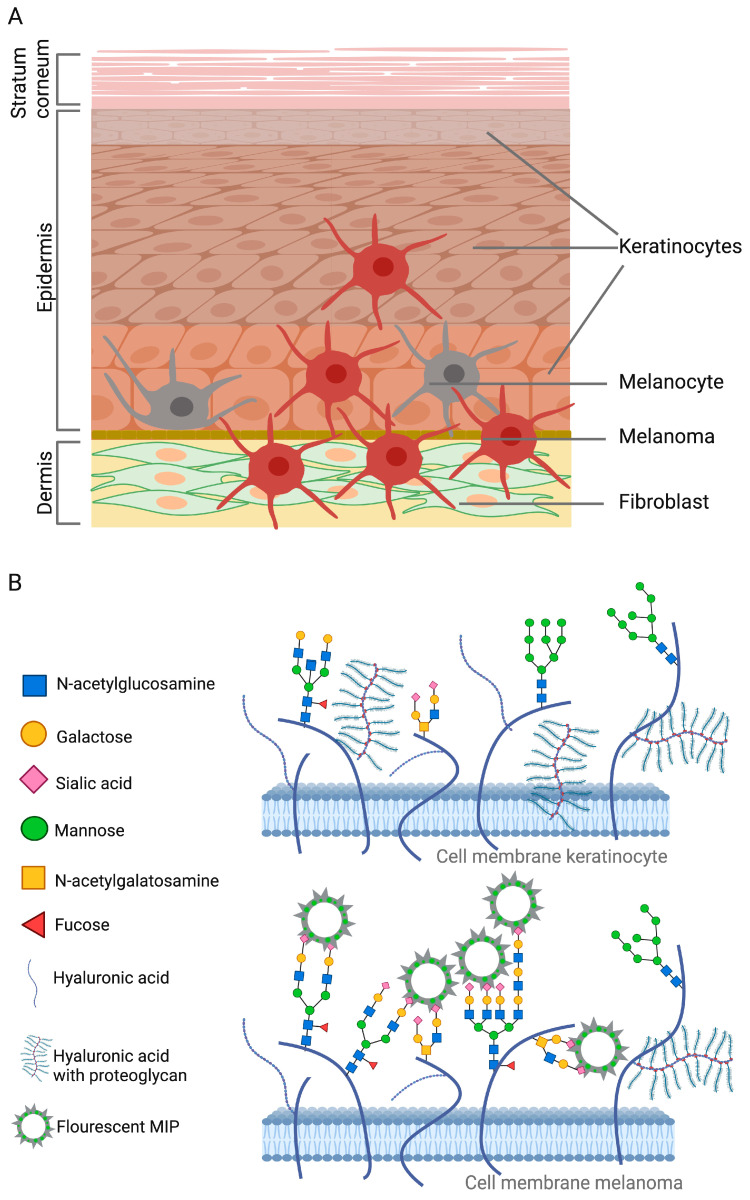
(**A**) The layers of the skin comprise the *stratum corneum*, with cornified flat keratinocytes (**upper**); the epidermis, with different stages of keratinocytes, melanocytes, and immune cells (**middle**); and the dermis, with mainly fibroblasts (**lower**). (**B**) The expression of monosaccharides and glycosaminoglycans is illustrated on the cell membranes of keratinocytes (**upper**) and melanoma cells (**lower**). Since the binding of SA-MIPs to keratinocytes was absent in the unpublished study, the cell membrane of this cell type is proposed to express more glycosaminoglycans than monosaccharides. On the other hand, melanoma cells are proposed to express high amounts of monosaccharides since these cells showed good SA-MIP binding.

SA-MIPs targeting skin cells have shown promising results. The skin is composed of the epidermis, which consists of epithelial tissue, and the dermis, which is composed of connective tissue. Keratinocytes of different maturation stages can be found together with Langerhans cells and melanocytes beneath the outermost layer of the epidermis, the *stratum corneum* [52] (Figure 2A). Melanoma can be initiated after a number of cell divisions and bypassing senescence (aging of cells) [53]. The dermis comprises loose connective tissue and a reticular layer of dense connective tissue, which contains fibroblasts responsible for the production of collagen, elastin, and glycosaminoglycans.

In a recent preliminary study, four different cell lines were subjected to fluorescent SA-MIP binding (unpublished). Interestingly, the two analyzed human keratinocyte cell types, A431 and Hek-n, did not show any binding to the SA-MIPs, as determined using flow cytometry. In contrast, a human melanoma cell line (A2058) and a mouse fibroblast cell line (L929) showed binding to the SA-MIPs (Figure 2B). The lower expression of SA in the keratinocyte population may be explained by the different expression or localization of SA on the cell types in the skin.

Indeed, MIPs targeting glycosaminoglycans have recently been developed. Hyaluronan (HA) is a non-sulfated glycosaminoglycan and a major extracellular matrix component. Kunath et al. used the monosaccharide glucuronic acid, which is a part of HA, as a template for molecular imprinting [54]. HA forms an important structural component of the extracellular matrix, acting as a scaffold for macroproteins, which decorate the HA chains [55]. The large amounts of water bound by HA are critical in maintaining adequate hydration within the skin, promoting both the physiological function of the skin and the maintenance of cosmetic skin quality. Glucuronic acid is part of the glycocalix or intercellular matrix, which is mainly found as a component of HA. However, other approaches have also been utilized for MIP synthesis, with glucuronic acid as the template and keratinocytes as a target [42,44,56]. Studies have included comparisons of SA and HA using MIPs; they have also compared live and fixed cells and MIP specificity by enzymatic cleavage and the use of non-imprinted MIPs.

Typically, MIPs developed for targeting also have imaging functionalities. MIPs designed for the tracking of glycans incorporate luminescent signals, which can be exploited for the imaging of fixed and living cells. The luminescent signal can come from different sources. For instance, Panagiotopoulou et al. compared the performance of fluorescent MIPs prepared by two different approaches: coating fluorescent quantum dots with an MIP layer and incorporating a polymerizable rhodamine B during the polarization step in the MIP preparation [42]. In a similar approach, FITC-doped silica particles were modified with an imprinted shell to target saccharides for the imaging of different types of cancer cells [45]. The imprinting was aided by the favorable interaction between the target and surface-immobilized boronic acid moieties. Boronic acid was also used for the preparation of gadolinium-doped silicon MIPs that could also be used for magnetic resonance imaging (MRI) [57]. Fluorescent monomers were used for MIP imprinting on core–shell polystyrene–silica particles targeting SA. Both flow cytometry studies and confocal microscopy studies showed that the binding pattern for MIPs varied between different cancer cell lines, and they attributed this variation to different patterns in SA expression or cell morphology [48]. In order to use more biocompatible materials, researchers have used carbon dots as a fluorescent core for MIPs. Confocal microscopy studies of carbon dots (MIPs) targeting an epitope of hyaluronic acid have shown that MIPs could differentiate between the tumor and healthy cells. Moreover, cytotoxicity was low [56]. Jiang et al. prepared dual fluorescent MIPs for the imaging of cancer cells. In this case, the fluorescence signal from the carbon nano-dot core and the fluorescence signal from the MIP coating film were detected in two different detection channels [58]. In other studies, epitopes of membrane proteins expressed in tumor cells, such as CD59 [59], or human epidermal growth factor receptor (EGFR)-2 [60] have been used as templates for silica-based MIPs.

(b) Drug delivery

Targeting and delivering drugs close to the site of the tumor in vivo is a challenge. Canfarotta et al. investigated the possibility of targeting the EGFR commonly overexpressed on many tumors with doxorubicin (DOX), a chemotherapeutic drug that is used to treat various types of cancer. DOX interferes with the DNA in cancer cells and prevents them from dividing. Canfarotta et al. found that the approach was selective and induced cell death in the targeted cancer cells [61]. There are a few studies of MIPs in drug delivery systems in vitro—for instance, with cytostatic drugs such as DOX. This drug is typically administered systematically and, thus, has many side effects. Therefore, targeting cytostatic drugs to tumor cells is preferred. MIPs developed for breast cancer treatment with sensitivity to an external magnetic field were studied as a device for controlled release, thereby for the delivery of DOX [62]. DOX-imprinted polydopamine used in a breast adenocarcinoma-bearing mouse model showed enhanced DOX uptake in tumor tissue and lower concentrations of DOX in kidney and liver tissue in groups treated with DOX-imprinted polydopamine and with a magnetic field, compared to groups of mice treated with free DOX and DOX-imprinted polydopamine without the magnetic field. Another approach to targeting tumors involving MIPs and DOX was demonstrated by Qin et al. [63], who used two different templates for imprinting: DOX and the epitope for the p32 protein, a receptor that can be upregulated in cancer. Breast cancer cells with upregulated p32 expression were injected subcutaneously in nude mice. The tumor-bearing mice were then injected with fluorescent MIPs (FMIPs) imprinted with DOX and the epitope for p32. The tumor volume was reduced in mice that received intravenous injections of FMIPs and DOX in comparison to animals injected with a control substance and free DOX. Qin et al. also compared intravenous and intra-tumoral injections to further analyze the targeted effect of FMIP imprinted with DOX and p32. No damage by toxicity on the heart, liver, spleen, lung, or kidney could be observed. Similar results were shown by Peng et al. [60], who used the CD59 epitope instead of p32. Hashemi-Moghaddam et al. also used epitope imprinting together with DOX for the delivery of DOX to HER2-expressing ovarian cancer cells in vivo [64]. They found a reduction in tumor growth in mice treated intravenously with MIPs imprinted with DOX and HER2. In comparison, MIPs imprinted with only DOX and free DOX could not reduce the tumor growth in the same experimental system. The uptake of DOX was measured and shown to be significantly higher in the tumors treated with DOX and HER2, demonstrating specific uptake through the epitope. Epitope imprinting provides significant advantages over protein imprinting when constructing MIPs, including reduced costs, the preservation of structural and functional properties, enhanced selectivity and specificity, and compatibility with various synthetic conditions.

(c) MIP-based biosensors in cancer

MIPs have been intensively studied for the detection of cancer biomarkers through electrochemical or optical sensing. A comprehensive review with a greater focus on sensor preparation can be found in Pilvenyte et al. [65]. Common biomarkers for MIPs used in cancer detection are prostate-specific antigen (PSA), HER-2 in breast cancer, CA-125 in epithelial cancer, and α-fetoprotein (AFP) in liver cancer. However, determining low concentrations of biomarkers in complex matrices is technically challenging. The reviewed studies used MIP-based biosensors to assess natural or artificial samples such as blood, serum, plasma, or urine. The review outlined the molecular imprinting technology and MIP-based sensor creation principles and discussed the analytical signal determination methods and the nature and chemical structure of the imprinted polymers [65]. There is an urgent need for the development of tests and assays to help improve cancer diagnostics and treatment. MIP-based sensors can be a faster and cheaper alternative to the laboratory-based assays that are used today. The latest achievements in the biosensing of cancer biomarkers using MIPs are classified below based on the biomarker/cancer type and summarized in Table 1.

- Prostate cancer and PSA

PSA is widely used in screening and diagnosing prostate cancer, although not without debate. The standard methods for PSA screening deploy immunoassays, like the ELISA. These methods are highly sensitive and specific for the detection of PSA, but they are also expensive because they require specific natural antibodies and special handling and storage [66]. In addition, the great affinity between the antibody and its antigen makes it impossible to dissociate these two biological components after binding. This feature limits PSA assays to a single-use application. Biosensors have emerged as an alternative to some ELISA methods, serving as an attractive tool for quick and local clinical analysis. Studies have reported several biosensors for PSA in the pg/mL range (Table 1) [66].

Technological advancement has also led to the development of optical sensors allowing the quantification of PSA. For instance, Turan et al. developed a combined MIP–antibody sensor that selectively detects PSA. The MIP magnetic particles have the function of targeting PSA, while the binding of antibody-modified gold nanoparticles used for surface-enhanced Raman spectroscopy (SERS) detection provides a measurable signal [67]. Another study analyzed surface plasmon resonance (SPR) detection by using micro-contact PSA-imprinted sensors [68]. Interestingly, the authors used the system to screen ten clinical serum samples for PSA content and reported that the assay showed 98% consistency with commercial ELISA methods.

Furthermore, electrosensors have been widely explored for the detection of PSA. Electrosensors based on imprinted PSA on graphene oxide were tested for the detection of PSA in serum [66] and, in a more biological context, in the culture medium of the prostate cancer cell lines PC3, LNCaP, and PNT2 [69]. In the second article, the cell culture medium was collected after different time points, and the PSA concentration was determined with the electrosensor. The binding of the protein was performed with a hydrolyzable amide bond, and, to improve the imprinting process, polar monomers that interacted with PSA were added during the imprinting process. The sensors were tested for repeated measures and showed similar responses for up to two months of use. Moreover, the performance of the electrosensors was similar to that of commercial ELISA kits. In a related approach, sensors were prepared using MnO_2_-modified carbon nanotubes. The presence of the MnO_2_ nanoparticles helped to maintain the conductive properties of the carbon material [70,71].

Yazdani et al. aimed to develop a robust biosensor for the quick diagnosis of prostate cancer by imitating the current antibody-based detection [72]. The authors presented a PSA biosensor based on molecularly imprinted electropolymerized polypyrrole. The MIP biosensors exhibited an improved limit of detection value compared to similar available techniques [72]. In another approach, a thiolated DNA aptamer with an established affinity for PSA was used in a complex as the template for imprinting [73]. The authors hypothesized that the imprinting around the aptamer helped to “lock” the aptamer in an optimal binding position to improve the sensitivity to the target. Thereafter, they used electrochemical impedance spectroscopy to evaluate the binding to the apta-MIP surface. However, it is not completely clear whether imprinting leads to higher affinity due to the creation of specific sites on the polymeric film or whether the polymerization around the aptamer facilitates the specific binding between the aptamer and the target. Moreover, the effect of post-imprinting modification on the specificity of a MIP sensor was explored by Matsumoto et al. [74]. By blocking low-specificity recognition cavities after imprinting, they found that the overall specificity of the material increased, although the total amount of bound PSA decreased. Another study utilized a dual-modality sensor based on MIPs and a nanostructured biosensing layer to simultaneously detect two biomarkers—PSA and myoglobin—in human urine and serum samples by impedance spectroscopy [75]. The results obtained from the dual detection and ELISA were in good agreement.

- Carcinoembryonic antigen

Carcinoembryonic antigen (CEA) is a glycoprotein produced during fetal development and is therefore absent in healthy adults. However, it is expressed in the following cancer tissues: colorectal, breast, ovarian, lung, gastric, bladder, and pancreatic [76]. CEA has been directly detected on electrosensors prepared via the in situ electropolymerization of a functional monomer in the presence of CEA. One study attempted to create self-powered sensors by combining the electrode with the sensing film with photovoltaic cells [77]. The study aimed to increase sensor suitability for point-of-care applications. However, the analytical performance of the integrated system was impaired compared to the performance of the imprinted electrode alone. In other approaches, MIP binding has been combined with detection aided by optical tags. Some optical devices use Raman spectroscopy for the detection of CEA with a pseudo-immune-sandwich assay. Zhou et al. targeted CEA in an immune sandwich between a molecularly imprinted film on a gold nanoparticle-modified glass slide and a molecularly imprinted silver nanotag as a Raman reporter [78]. The imprinted target for the film (an epitope from the C- or N-terminal of CEA) and for the nanotag (glycoproteins digested from the target protein) ensured improved specificity. In a study by Lin et al. [79], the target protein was immobilized between an imprinted film on a SERS sensor that also contained antibodies against the target protein and a reporter dopamine-coated gold nanoparticle modified with antibodies. The authors pointed out that even though the performance was satisfactory, the synthesis procedure must be simplified for clinical use.

Another study prepared molecularly imprinted magnetic nanoparticles for specific binding to different glycoproteins, including CEA. The amount of bound protein was detected upon its extraction from the medium via a fluorescence probe, which used boronate’s affinity to the trapped protein. The system showed similar performance to ELISA methods, but the preparation of the MIPs and fluorophore remains chemically challenging [80].

- Breast cancer

Carbohydrate antigen CA15-3 and EGFR-2/HER2 have been used as templates to fabricate electrosensors for the detection of breast cancer biomarkers. The standard strategy is the electropolymerization of a functional monomer on different electrodes and the indirect determination of the target concentration due to the displacement of a redox probe [81,82,83,84]. Ribeiro et al. used a polymerizable dye, toluidine blue, which is commonly used as an electronic mediator, to create a polymeric film that yielded a sensor with enhanced conductivity [85]. The sensors evaluated in serum showed recovery of 70–100%, but the recovery was lower for detection in saliva, equaling 62–76%. You et al. recently designed and evaluated a system for the detection of the breast cancer susceptibility gene BRCA-1 in human serum samples [86]. Their results showed high sensitivity and selectivity based on the specific recognition of MIPs and signal amplification using SiO_2_@Ag nanoparticles.

- Other types

In addition to the aforementioned cancer detection systems, volatile organic compounds have been proposed as cancer biomarkers that can be detected by non-invasive tests. A few studies have developed sensors for the detection of volatile aldehydes with a common approach—namely, drop casting pre-imprinted polymeric nanoparticles and gold nanoparticles or carbon nanotubes. The nanostructures embedded in the sensors enhance the film conductivity and hence the sensor signal [87]. A point-of-care device has also been designed to detect multiple volatile biomarkers based on the electropolymerized molecularly imprinted film. The device can detect several prospective lung cancer biomarkers at the ppt level [88].

**Table 1:** Overview of the characteristics and performance of biosensors used in cancer applications.

Biomarker	Format of the MIP Sensor	Method of Imprinting	Detection Principle	Limit of Detection (LOD)	Ref.
PSA	magnetic MIP particles combined with PSA-antibody-labeled AuNP	surface imprinting (core–shell)	SERS	0.9 pg/mL	[67]
PSA	film on a gold SPR sensor chip	microcontact imprinting	SPR	91 pg/mL	[68]
PSA	film on graphene oxide sheets	surface imprinting after template immobilization	potenciometry	2∙10^3^ pg/mL	[66,69]
PSA	film with MnO_2_-particle-modified CN on a graphite electrode	drop casting and surface imprinting	voltammetry	3.04∙10^−3^ pg/mL	[70,71]
PSA	film on gold screen-printed electrode	electropolymerization	voltammetry	2 pg/mL	[72]
PSA	film on gold electrode/anchoring aided by a DNA aptamer	electropolymerization	EIS	10 pg/mL	[73]
PSA (+Mb)	film on modified SPR gold chip	surface imprinting	SPR	5.4∙10^3^ pg/mL	[74]
PSA	film on gold screen-printed electrode	surface imprinting	EIS	0.83∙10^3^ pg/mL	[75]
CEA	film on fluorine-doped tin oxide glass	electropolymerization	voltammetry	10 pg/mL	[77]
CEA	film on a glass coated with AuNP and surface-imprinted AgNP	surface imprinting	SERS	10 pg/mL *	[78]
CEA	magnetic iron nanoparticles with boronate groups	BAC-oriented surface imprinting	fluorescence	1.2∙10^−3^ pg/mL	[80]
CEA	gold/silver core–shell particles embedded in MIP film on a gold-coated microarray substrate	BAC-oriented surface imprinting	SERS	64∙10^−3^ pg/mL	[79]
HER-2	gold nanostructures in MIP film on laser-scribed graphene	electropolymerization	voltammetry	0.43∙10^3^ pg/mL	[81]
HER-2	film on gold screen-printed electrode	electropolymerization	voltammetry	1.6∙10^3^ pg/mL	[82]
CA 15-3	film on gold screen-printed electrode	electropolymerization	voltammetry	1.5 U/mL	[83]
CA 15-3	AuNP in a MIP matrix on a paper-based electrode	electropolymerization	voltammetry	1.16 U/mL	[84]
CA 15-3	poly-toloudine blue polymer on a gold screen-printed electrode	electropolymerization	voltammetry	<0.10 U/mL	[85]
BRCA-1	AuNP embedded in an MIP film on a glass carbon electrode	surface imprinting	voltammetry	2.53 fM	[86]
VOC	MIP particles on AuNP and drop-cast on an interdigitated electrode	precipitation polymerization	voltammetry	1.1 ppm	[87]

PSA = prostate cancer antigen, Mb = myoglobin, CEA = carcinoembryonic antigen, HER-2 = human epidermal growth factor receptor 2, CA 15-3 = cancer antigen 15-3, BRCA-1 = breast cancer type 1 susceptibility protein, VOC = volatile organic compounds, AuNP = gold nanoparticles, AgNP = silver nanoparticles, CN = carbon nanotubes, BAC = boronate affinity-controllable, EIS = electrochemical impedance spectroscopy, SERS = surface-enhanced Raman spectroscopy, SPR = surface plasma resonance. * Recalculated from data in reference [77] with a molecular weight of 180 kDa for CEA.

## 5. Neurodegenerative Diseases

Several studies have explored the use of MIP sensors to detect neurotransmitters such as dopamine. However, dopamine levels in biological fluids such as cerebrospinal fluid or blood are not considered suitable biomarkers for the diagnosis of neurodegenerative diseases [67]. Other studies have focused on extracting and detecting proteins or peptides that are actually used as biomarkers for the diagnosis neurodegenerative diseases, such as Alzheimer’s and Parkinson’s disease.

The levels of the different isoforms of the peptide β-amyloid are a valuable marker for the diagnosis of Alzheimer’s disease. Accordingly, β-amyloid 1–42 is currently used as a biomarker for Alzheimer’s disease. Urraca et al. investigated a method to enrich these peptides and extract them in serum using MIPs [67]. MIP sensors containing carbon nanotubes were developed for the detection of β-amyloid 1–42. In one study, a composite formed by layers of two-dimensional inorganic compounds (MXenes) and multiwall carbon nanotubes was drop-cast on a carbon glassy electrode. After this, pyrrole in the presence of the target peptide was electropolymerized to create the specific binding sites. The study found that the composite provided good conductivity and surface area due to the MXenes, as well as stability due to the carbon nanotubes [89]. In a second study, imprinting was performed on the surfaces of single-wall carbon nanotubes. Before polymerization, the carbon nanotubes were oxidized to provide reactive points for the covalent immobilization of β-amyloid 1–42. The imprinting step yielded imprinted carbon nanotubes that were afterward embedded in PVC membranes deposited on a graphite electrode [90]. Both studies proved good selectivity for β-amyloid 1–42 in plasma. However, the selectivity of these sensors for β-amyloid 1–40, which differs from β-amyloid 1–42 only by two amino acids on the C-terminal of the sequence, was not explored or discussed. This is certainly a drawback in the sensor validation, taking into consideration that it is the concentration of the 1–42 variant or the ratio of β-amyloid 1–42/1–40 that is linked to the progression of the disease and makes it useful as a biomarker [91].

Another relevant protein in Alzheimer’s disease detection is p-Tau. One study employed an electrochemical biosensor prepared by electropolymerization in the presence of p-Tau-441 as the template [92]. The performance of the corresponding imprinted and non-imprinted electrodes was evaluated by electrochemical impedance spectroscopy, and it showed good selectivity in the serum samples. Thus, the electrochemical biosensor was considered a potential tool for the screening of the Tau protein onsite and an attractive complement to clinically established methodologies. However, the authors reported diminished performance in serum compared to buffer controls, probably due to the competitive binding of other serum components. Interestingly, one unusual application for MIPs is the detection of volatile compounds in breath, which have been identified as possible biomarkers for Alzheimer’s disease [93].

In another neurodegenerative disorder, Parkinson’s disease (PD), the protein α-synuclein is a well-studied biomarker [94]. For instance, magnetic MIP nanoparticles were tested for the binding and extraction of α-synuclein from cell cultures [95]. A 15-amino-acid peptide associated with α-synuclein aggregation behavior was used as a template for the imprinting. The study used immunostaining to observe the removal of α-synuclein from protein-expressing cells. Interestingly, another study used three different short peptide sequences of α-synuclein as templates for sensor construction by epitope electropolymerization molecular imprinting [95]. To test the MIPs in a relevant microenvironment, the authors cultured midbrain organoids and idiopathic PD organoids. The culture medium from the organoids was measured along with MIP-based electrodes. Together with fluorescence studies, they suggested that α-synuclein aggregated in idiopathic organoids [95]. In a similar approach, Ma et al. imprinted the whole protein on an electropolymerized MIP film [96]. The attachment of the protein to the surface during sensor preparation was aided by cross-linking between the protein and glutaraldehyde. Another biomarker for PD is DJ-1, which was also used as a template in sensor fabrication [96,97]. DJ-1 sensors were tested for PD detection in doxycycline-induced NA2 cells.

## 6. Cardiovascular Diseases

MIP sensors developed to detect biomarkers for cardiovascular diseases focus mainly on myoglobin, angiotensin, and troponin T and I (TnT and TnI). The detection of cardiac biomarkers such as TnT is important for both the early diagnosis of myocardial infractions and the utility of using high-affinity MIPs instead of commercial antibodies.

To determine cardiovascular biomarkers, Moreira et al. used myoglobin-imprinted films by electropolymerizing *o*-aminophenol around a protein layer previously absorbed into gold [98]. A short measuring time, reusability, and low detection limit were shown together with good selectivity towards myoglobin. Moreover, Phonklam et al. found a suitable candidate for a point-of-care device measuring cardiac TnT with an electrochemical MIP sensor [99]. In this study, the thickness of the polymer layer was controlled by the cycles of electropolymerization. A small number of cycles led to a film that may not have whole-formed cavities for the template, while a too high number of cycles could hinder electron transfer. The sensors showed similar performance to a gold-standard ELISA method used in spiked human plasma. Similar sensors have been fabricated with different combinations of support and polymer [100,101,102]. In one study, SPR was proposed for the real-time testing of cardiac injury by monitoring TnT released from cardiac tissue into the bloodstream [103]. The study also highlighted the importance of epitope selection. Of the four tested epitopes, only one located on the C-terminal of the protein successfully imprinted specific sites for the binding of the whole protein. Polynorepinephrine has also been used in an MIP biosensor [104]; interestingly, the study combined SPR detection and polynorepinephrine-based imprinting to detect TnI. However, the sensitivity of the sensors was not satisfactory, and an amplification step with an enzyme-labeled antibody was included [104].

In a slightly different approach, MIP particles were pre-imprinted before deposition on the sensing surface. Nano-MIP particles attached to screen-printed graphite electrodes were utilized for the thermal detection of cardiac TnT [105]. Nano-MIPs imprinted with a short epitope (10 amino acids) for TnI were immobilized on an SPR-modified gold chip. Although the chip preparation comprised more steps than many electropolymerization methods, the chip’s measuring, removal, and conditioning took under 15 min. However, the sensor was not tested with biological samples [106].

Other work has focused on the binding between target biomarkers and MIPs as a pre-step before analysis. MIPs have been used as an enrichment step for MALDI-TOF mass spectrometry measurements of TnI peptides [107]. Angiotensin II is involved in hypertension, and its recognition from human serum has been studied by immobilization using molecularly imprinted spongy columns followed by elution and UV–vis detection [108].

Lipoprotein levels have often been used to diagnose coronary heart disease. Chunta et al. investigated the detection of different types of lipoproteins using films of MIPs on a quartz crystal microbalance (QCM). A thin film of a pre-polymerized mixture of porogen, a functional monomer, a cross-linker, and an initiator was created by spin coating on the quartz crystal and then adding the template and low-density/high-density lipoprotein and performing the final polymerization. Sensor performance was tested against spiked human serum or artificial human serum with controlled concentrations of low-density and very low-density lipoproteins. MIP QCM showed better performance than other QCM-based sensors and a similar detection range compared to clinical methods without the hazard of sample pre-treatment. The authors also showed the possibility of the simultaneous detection of different types of lipoproteins. However, the time for the cleaning and regeneration of the sensors after measurement (approximately 30 min) was not discussed [109,110,111].

## 7. COVID-19

Following the outbreak of COVID-19, different diagnostic sensors have been proposed with an emphasis on point-of-care use. Two different approaches have been used to prepare electrochemical sensors (and one optical sensor): modifying the electrode with a molecularly imprinted thin film [112,113,114,115] or modifying it with MIPs synthesized by solid-phase synthesis [116,117]. In some studies, the SARS-CoV-2 nucleoprotein, the spike S protein subunit S1, and the receptor-binding domain have been used as templates for imprints on thin-film gold electrodes or gold screen-printed electrodes. Sensors on the different platforms rely on the signal of a redox probe that is displaced by the binding of the target molecule to the imprinted sites. The nucleoprotein and S1 subunit sensors showed specificity when compared with other antigen recognition sites. However, the study using biological samples was not comprehensive enough to show selectivity against other viruses [112,113]. The sensor for the receptor-binding domain subunit has not been tested in biological samples [118]. An electropolymerized molecularly imprinted film on platinum electrodes was studied to detect the spike S protein, but results in biological samples from infected patients are lacking [114]. A similar electropolymerization approach was followed in another study, but the template mimicked the whole virus. The electrode did not show a broad detection range, and the testing of a small pool of patient saliva samples showed 75% consistency compared to established methods for COVID-19 diagnosis (e.g., loop-mediated isothermal amplification) [115].

Other electrochemical sensors detecting SARS-CoV-2 took a different approach: instead of MIP films, solid-phase synthesized MIP particles were electro-grafted on a graphite electrode surface for heat-transfer-based measurements. In this case, the template was an epitope of the receptor-binding domain of SARS-CoV-2 instead of the whole virus or protein. Compared to commercial antigen tests, the sensor showed an improved detection limit; moreover, it was comparable to the reverse transcription polymerase chain reaction analysis in a study of 14 patient saliva samples, where seven were COVID-19-positive and seven were negative [117]. In addition, solid-phase particles were used to synthesize MIP particles to prepare an optical sensor. As in the previous work, the whole virus was imprinted on the particles that were attached to the gold SPR chip, but sensor validation in biological samples was not performed [115].

## 8. Renal Disease

MIP sensors for renal disease focus on detecting creatinine as a biomarker. An early report presents a sensor constructed by assembling pre-imprinted magnetic Fe_3_O_4_–polyaniline nanoparticles [119]. The magnetic character of the particles allowed magnetic deposition on the electrode, followed by electropolymerization. Another electrosensor used imprinted polydopamine on conductive graphene nanoplatelets that were drop-cast on an electrode. The high conductivity of the cast film provided a strong signal and high sensitivity [120]. Optical sensors have also been developed for the detection of creatinine. In one application, a gold electrode for SPR was modified via the photopolymerization of N-methacryoryl-(L)-histidine methyl ester in the presence of creatinine bound to the functional polymer via copper ions [121]. In another case, an optical fiber with long-period grating was used to evaluate creatinine recognition. The optical fiber was modified first through the layer-by-layer deposition of a mesoporous film of PDDA/SiO_2_ to increase the surface area of the fiber and subsequently through the deposition of a titanium-imprinted film. Changes in the refractive index of the fiber were used to determine the specific binding of creatinine to the imprinted cavities [122].

## 9. Discussion and Conclusions

The field of molecular imprinting and its applications in biochemistry, biology, and medicine have been intensively developed in the last twenty years. This is emphasized by all the publications that have emerged in the field. MIP applications for biology and medicine are currently widely explored and developed at a small-scale level. The synthetic recognition sites of the MIPs can be tailor-made to function in analytics, diagnostics, and drug delivery systems, which will greatly improve personalized medicine. In this review, we have shown that established disease biomarkers are used in a variety of biosensors, optical and electrochemical sensors, imaging, and drug delivery. Since MIPs are chemically designed, their fabrication is a complex process that involves choosing multiple parts, such as the format of the MIPs, direction of synthesis, template, monomers, and fluorophores.

With the wide range of formats to use depending on the application, MIPs have increased in popularity. The possibilities to synthesize MIPs and their components are vast. We have described different MIP formats, from a simple polymeric film on a surface to more intricate systems composed of a nanoparticle-decorated film and core–shell molecularly imprinted nanoparticles. This allows MIPs to be designed towards almost any desirable target. The MIP technique enables the design of materials mimicking natural antibodies. Moreover, the analytical performance of MIP-based sensing is similar or, in some cases, even better than that of antibody-based assays. In some applications, antibodies are the first choice today, but they may be exchanged for excellent-performing MIPs. Natural antibodies often fail to perform against small or simple antigens due to the lack of specificity and sensitivity. Here, MIPs can be further developed and refined to be the ultimate option for biosensing.

Despite the numerous MIP applications found in the literature, there are still few commercial MIPs available. Issues such as scalability and reproducibility could be an obstacle to creating a broader range of MIP-based systems. The possibilities to synthesize MIPs and the precise methods for each application still need to be refined. A drawback for future commercial development is the lack of a universal protocol for MIP synthesis. Moreover, the MIP assays must be validated with clinically relevant samples and compared to established methods to ensure their adequacy in the clinical context. A longer path to clinical use is expected for MIP systems intended to be used for in vivo application. Here, more knowledge about the drug release, biodegradability, toxicity, distribution, and clearance of MIP systems is needed at the basic research level before employing suitable targeting and drug delivery systems for in vivo usage.

To conclude, the sensitivity of the different MIP-based sensors is promising but depends on many variables in both the synthesis and analysis process. Future work in the field will likely focus on developing the separate parts of the fabrication process and on MIPs’ increased applicability in vivo, subsequently enhancing their usability in the diagnostics and treatment of various diseases.

## Data Availability

No new data were created.
